# The CHA2DS2-VASc Score as an Early Predictor of Graft Failure After Coronary Artery Bypass Surgery

**DOI:** 10.7759/cureus.22833

**Published:** 2022-03-04

**Authors:** Omer Tasbulak, Anil Sahin

**Affiliations:** 1 Cardiology, University of Health Sciences, Mehmet Akif Ersoy Thoracic and Cardiovascular Surgery Training and Research Hospital, Istanbul, TUR

**Keywords:** graft, graft failure, graft patency, coronary artery bypass surgery, cha2ds2-vasc

## Abstract

Background and objective

Graft patency is one of the major concerns after coronary artery bypass graft (CABG) surgery. The CHA_2_DS_2_-VASc [congestive heart failure, hypertension, age ≥75 years, diabetes mellitus (DM), stroke or transient ischemic attack (TIA), vascular disease, age 65 to 74 years, sex category] score is a tool that was developed to predict the risk of thrombotic events in patients with atrial fibrillation (AF). In this study, we evaluated the use of the CHA_2_DS_2_-VASc score as a simple tool for predicting graft failure (GF) among patients who underwent CABG surgery.

Methods

In this retrospective case-control study, a total of 280 patients were enrolled after applying the exclusion criteria. Angiograms were analyzed by using the QCA software system (Pie Medical Imaging, Maastricht, The Netherlands) for each patient. A graft was described as failed if it had 70% or more stenosis or was completely occluded. Patients were classified into two groups: group one included patients without GF (GF-N) and group two included patients with GF (GF-Y). Thereafter, the CHA_2_DS_2_-VASc risk score was calculated for each patient.

Results

In our cohort, 136 patients had GF (GF-Y group) and 144 patients did not have GF (GF-N group). GF-N and GF-Y patients had their angiography performed 100.31 ± 8.04 and 103.49 ± 8.41 months after CABG, respectively. GF-Y group had a significantly higher rate of DM, hypertension, and heart failure with reduced ejection fraction (HFrEF). GF-Y group had higher CHA_2_DS_2 _(GF-N group: 1.47 ± 0.91 vs. GF-Y group: 2.57 ± 1.17, p=0.0001) and CHA_2_DS_2_-VASc score (GF-N group: 2.80 ± 1.11 vs. GF-Y group: 4.15 ± 1.25, p=0.0001). Analyses showed that only CHA_2_DS_2_-VASc was an independent predictor of GF while other parameters including DM, hypertension, HFrEF, creatinine, and CHADS_2 _were not found to be independent predictors of GF. A CHA_2_DS_2_-VASc score of >3 predicted GF with a sensitivity of 65.44% and a specificity of 74.31%.

Conclusions

The CHA_2_DS_2_-VASc score might be used as a feasible and simple method to predict the risk of GF after CABG surgery.

## Introduction

Coronary artery bypass graft (CABG) is the treatment method of choice in patients with the left main coronary artery (LMCA) or 3-vessel disease, especially in those with diabetes mellitus (DM) [1]. CABG is typically performed on the cardiopulmonary bypass, which provides surgeons a good visualization and motionless field, thereby allowing them to perform the procedure more effectively. Because of the necessity of a vascular intervention that affects outcomes and re-hospitalization, graft patency and longevity are one of the major concerns after CABG [2-4]. The known mechanisms associated with graft failure (GF) are thrombosis, neo-intimal hyperplasia, and atherosclerosis [5,6]. The CHA_2_DS_2_-VASc [congestive heart failure, hypertension, age ≥75 years, diabetes mellitus (DM), stroke or transient ischemic attack (TIA), vascular disease, age 65 to 74 years, sex category] score is a tool that was developed from CHADS_2_ to predict the risk of thrombotic events in patients with atrial fibrillation (AF) [7]. The difference between CHA_2_DS_2_-VASc and CHADS_2_ lies in the fact that apart from the common parameters for both tools, the former also factors in the history of vascular events, the presence of peripheral artery disease and plaque in the aorta, and sex category. The score mainly assesses the comorbidities in patients that may lead to a high risk of thrombosis. However, with further studies, the score has not only come to indicate the thrombogenic risk in patients with AF but a strong relationship has also been observed between cardiovascular adverse events and the score [8].

In this study, we evaluated the use of the CHA_2_DS_2_-VASc score as a simple tool for predicting the incidence of GF among patients who underwent CABG surgery.

## Materials and methods

In this retrospective case-control study, the subjects were selected from among the 1,875 consecutive patients who underwent isolated CABG surgery between October 2009 and December 2011. Patients over 18 years of age with exertional angina or exertional dyspnea, with ischemic origin ventricular arrhythmias, and patients with newly developed segmental wall motion defect, with a decrease in ejection fraction during follow-up, were also included in the study. In addition, those who later underwent coronary angiography (CAG) due to stable angina pectoris refractory to medical treatment or acute coronary syndrome were also included in the analysis. In patients presenting with stable angina pectoris, CAG was indicated by the objective evidence of ischemia, including positive exercise stress testing or positive radionuclide noninvasive test [9]. Patients with a prior history of severe valvular heart disease, congenital heart disease, history of end-stage renal failure, liver failure, malignancy, emergent CABG surgery, lack of regular follow-up, and those who were non-adherent to their medical treatment were excluded [10]. Patients who received oral anticoagulation following surgery and patients whose preoperative CHA_2_DS_2_-VASc score could not be calculated because of insufficient data were also excluded. After the exclusion criteria had been applied, a total of 280 patients were enrolled in the present study.

Data regarding demographic factors, cardiovascular risk factors, smoking status, and physical examination on admission were obtained from our hospital database and patient files. All biochemical and hematological parameters were recorded before the CABG operation. Patients who had a diagnosis of heart failure with reduced ejection fraction (HFrEF) were evaluated based on their preoperative echocardiographic records. Informed consent was obtained from all patients. The study adhered to the ethical guidelines of the 1975 Declaration of Helsinki protocol and was approved by the Ethics Committee of our hospital.

Angiographic analysis

CAG graft study was performed through the femoral or radial artery using 6 Fr or 7 Fr sheaths. At least four and two images were obtained for the left and right coronary arteries, respectively. In addition, the left internal thoracic artery and each aortic anastomosis were selectively visualized. Aortic root injection was also administered if the vein bypass grafts could not be localized by graft or stump injection. All angiograms were evaluated by two interventional cardiologists blinded to the patients’ clinical and laboratory data and were measured by using a software system (QCA, Pie Medical Imaging, Maastricht, The Netherlands). Coronary scopic images were evaluated and the best views demonstrating the reference vessel in the longitudinal axis with minimal curvature in the scopic view were selected for analysis. QCA analysis was performed as detailed in the study by van der Zwet and Reiber [11]. After calibration, the scopic image was paused in a diastolic in which the graft was best filled with a flow. After calibration and tracking of the lesion, the system used automatic edge detection of vessels and provided the calculated parameters. GF was identified if it had stenosis greater than 70%. If a graft had less than 70% stenosis and the whole course of the graft was visualized, it was identified as patent. In sequential bypass grafts, each graft was accepted as an individual graft. Patients were classified into two groups: group one included patients without GF (GF-N), and group two included patients with GF (GF-Y).

CHA_2_DS_2_-VASc risk score

In our study, the CHA_2_DS_2_-VASc score was calculated by assigning 1 point each for the presence of congestive heart failure, hypertension, vascular diseases, DM, and female sex; 2 points for prior stroke or TIA; and 0, 1, or 2 points depending on age (age 65-74) [12]. Heart failure was defined as either any diastolic heart failure or a systolic ejection fraction of less than 40%; hypertension was defined as systolic blood pressure ≥140 mmHg, or diastolic blood pressure ≥90 mmHg or antihypertensive medication use; diabetes was defined as fasting blood glucose ≥126 mg/dL or the use of insulin or oral hypoglycemic agents [13].

Statistical analyses

Statistical analyses were performed using the SPSS Statistics software for Windows, version 24.0 (IBM, Armonk, NY). Numerical variables showing normal distribution were presented as mean ± standard deviation (SD), and categorical variables as percentages (%). Pearson chi-square and Fisher's exact tests were performed for categorical variables. While Student's t-test was used for comparing quantitative variables with normal distribution, the Mann-Whitney U test was used for comparing quantitative variables without normal distribution. Logistic regression analyses were used to determine the independent predictors of GF. Receiver operating characteristic (ROC) curve analysis was conducted to determine the optimal value of the CHA_2_DS_2_-VASc score to indicate GF in terms of sensitivity, specificity, and positive and negative predictive values. A p-value <0.05 was considered statistically significant.

## Results

A total of 280 patients [220 males (78.6%)] were included in the study. Demographic, clinical, and laboratory characteristics of the patients together with CAG characteristics and events are summarized in Table 1.

**Table 1:** Demographic, clinical, laboratory, and angiographic characteristics of the patients GF-N: patients without graft failure; GF-Y: patients with graft failure; SD: standard deviation; MI: myocardial infarction; PCI: percutaneous coronary intervention; PAD: peripheral arterial disease; HFrEF: heart failure with reduced ejection fraction; AF: atrial fibrillation; HDL: high-density lipoprotein; LDL: low-density lipoprotein; HbA1c: hemoglobin A1c; LAD: left anterior descending; LIMA: left internal mammary artery; D1: first diagonal artery; IM: intermediary artery; CX: circumflex artery; OM: obtuse marginal artery; RCA: right coronary artery; PDA: posterior descending artery; PL: posterolateral artery; CHADS_2_: congestive heart failure, hypertension, age ≥75, diabetes mellitus, stroke; CHA_2_DS_2_-VASc: congestive heart failure, hypertension, age ≥75 years, diabetes mellitus, stroke or transient ischemic attack (TIA), vascular disease, age 65 to 74 years, sex category

Characteristics	GF-N (n=144)	GF-Y (n=136)	P-value
Age, years, mean ± SD	58.52 ± 8.1	60.24 ± 7.96	0.074
Gender, female, n (%), male, n (%)	25 (17.36%)	35 (25.74%)	0.088
	119 (82.64%)	101 (74.26%)	
Smoking, n (%)	74 (51.39%)	85 (62.50%)	0.061
Diabetes mellitus, n (%)	64 (44.44%)	84 (61.76%)	0.004
Hypertension, n (%)	106 (73.61%)	131 (96.32%)	0.0001
Previous MI, n (%)	71 (49,31%)	69 (50,74%)	0,881
Previous PCI, n (%)	64 (44.44%)	56 (41.18%)	0.581
History of PAD, n (%)	11 (7.64%)	16 (11.76%)	0.242
History of cerebrovascular event, n (%)	20 (13.89%)	26 (19.12%)	0.238
HFrEF, n (%)	54 (37.5%)	66 (48.5%)	0.041
The time interval between operation and angiography, months, mean ± SD	100.31 ± 8.04	103.49 ± 8.41	0.092
CHADS_2_, mean ± SD	1.47 ± 0.91	2.57 ± 1.17	0.0001
CHA_2_DS_2_-VASc, mean ± SD	2.80 ± 1.11	4.15 ± 1.25	0.0001
Total cholesterol, mg/dl, mean ± SD	201.06 ± 52.92	207.49 ± 65.26	0.366
HDL, mg/dl, mean ± SD	39.63 ± 12.92	38.26 ± 10.32	0.330
LDL, mg/dl, mean ± SD	130.55 ± 42.48	131.54 ± 42.42	0.846
Triglyceride, mg/dl, mean ± SD	192.87 ± 91.17	209.61 ± 115.35	0.178
Glucose, mg/dl, mean ± SD	137.17 ± 56.44	143.07 ± 64.25	0.415
HbA1c, %, mean ± SD	6.8 ± 1.68	7.18 ± 2.08	0.099
Creatinine, mg/dl, mean ± SD	0.91 ± 0.23	1.05 ± 0.36	0.0001
Hemoglobin, g/dl, mean ± SD	13.58 ± 1.60	13.66 ± 1.74	0.701
Number of grafts, mean ± SD	3.19 ± 0.8	3.35 ± 0.8	0.101
LAD-LIMA, n (%)	137 (95.14%)	131 (96.32%)	0,625
LAD saphenous, n (%)	9 (6.25%)	16 (11.76%)	0,106
D1 saphenous, n (%)	68 (47.22%)	69 (50.74%)	0.557
IM saphenous, n (%)	14 (9.72%)	24 (17.65%)	0.053
CX saphenous, n (%)	32 (22.22%)	30 (22.06%)	0.974
OM saphenous, n (%)	87 (60.42%)	87 (63.97%)	0.540
RCA saphenous, n (%)	71 (49.31%)	68 (50%)	0.908
PDA saphenous, n (%)	40 (27.78%)	30 (22.06%)	0.269
PL saphenous, n (%)	4 (2.78%)	5 (3.68%)	0.670

Among the participants, 136 patients had GF (GF-Y group) and 144 patients did not have GF (GF-N group). There was no significant difference between groups in terms of age, gender, smoking status, history of myocardial infarction (MI), percutaneous coronary intervention (PCI), peripheral arterial disease (PAD), and cerebrovascular event. GF-Y group had a significantly higher rate of DM, hypertension, and HFrEF. GF-N and GF-Y patients had their angiography performed 100.31 ± 8.04 and 103.49 ± 8.41 months after CABG, respectively. There was no significant difference in terms of time intervals between surgery and angiography between the groups (p=0.092). GF-Y group had higher CHA_2_DS_2_ (GF-N group: 1.47 ± 0.91 vs. GF-Y group: 2.57 ± 1.17, p=0.0001) and CHA_2_DS_2_-VASc score (GF-N group: 2.80 ± 1.11 vs. GF-Y group: 4.15 ± 1.25, p=0.0001). In the laboratory parameters of the patients, there was no difference between the two groups except in creatinine levels. Creatinine levels were higher in the GF-Y group; however, both groups had creatinine levels in the normal range. There was no significant difference between the groups in terms of the number and location of grafts.

Logistic regression analysis was performed to identify factors predicting the existence of GF. Analyses showed that only CHA_2_DS_2_-VASc was associated with the presence of GF and was an independent predictor of GF while other parameters including DM, hypertension, HFrEF, creatinine, and CHA_2_DS_2_ were not found to be independent predictors of GF (Table 2).

**Table 2:** Logistic regression analyses for the investigation of independent correlates for graft failure HFrEF: heart failure with reduced ejection fraction; CHADS_2_: congestive heart failure, hypertension, age ≥75 years, diabetes mellitus, stroke; CHA_2_DS_2_-VASc: congestive heart failure, hypertension, age ≥75 years, diabetes mellitus, stroke or transient ischemic attack (TIA), vascular disease, age 65 to 74 years, sex category

	B	P	OR	95% CI for OR	
				Low	High
Diabetes mellitus	0.72	0.231	2.04	0.64	6.57
Hypertension	0.37	0.625	0.69	0.15	3.09
HFrEF	-0.03	0.290	0.97	0.93	1.02
Creatinine	0.39	0.689	0.68	0.10	1.57
CHADS_2_	0.15	0.793	1.16	0.38	1.55
CHA_2_DS2-VASc	0.82	0.045	2.28	1.02	5.09

The ROC curve analysis was performed to detect the optimal cut-off value of CHA_2_DS_2_-VASc in predicting GF. The area under the curve (AUC) for CHA_2_DS_2_-VASc was 0.779 (95% CI: 0.726-0.826). A CHA_2_DS_2_-VASc score of >3 predicted GF with a sensitivity of 65.44%, specificity of 74.31%, a positive predictive value of 70.61, a negative predictive value of 69.53, and a likelihood ratio of 2.55 (Table 3).

**Table 3:** ROC analysis of CHA2DS2-VASc score for graft failure CHA_2_DS_2_-VASc: Congestive heart failure, hypertension, age ≥75 years, diabetes mellitus, stroke or transient ischemic attack (TIA), vascular disease, age 65 to 74 years, sex category

CHA_2_DS_2_-VASc	AUC		SE			95% CI		
	0.779		0.028			0.726-0.826		
	Cut-off	Sensitivity		Specificity	PPV		NPV	LR (+)
	>3	65.44		74.31	70.61		69.53	2.55

## Discussion

Our data showed that DM, hypertension, HFrEF, increased creatinine levels, CHA_2_DS_2_ scores, and CHA_2_DS_2_-VASc scores were associated with early GF. All of the clinical comorbidities, which were found to be associated with GF, are components of CHA_2_DS_2_-VASc score and associated with increased ischemic risk and atherosclerotic process. The main finding of our study was as follows: even though components of CHA_2_DS_2_-VASc might not independently predict GF, CHA_2_DS_2_-VASc score independently predicted GF after CABG surgery. CHA_2_DS_2_-VASc score >3 significantly predicted early GF after CABG surgery.

CABG surgery is a preferred method of treatment for ischemic heart disease. In surgery, arterial or venous grafts are used. The development of degenerative atherosclerosis and thrombotic debris significantly contributes to the pathophysiology of graft occlusion [14]. Thrombosis (in acute period), neo-intimal hyperplasia (in subacute period), and atherosclerosis (in late period) contribute to the deterioration of graft after CABG surgery [15]. The main predisposing factor contributing to the deterioration of grafts is the age of the graft; preventive measures include matching and quality assessment of the native and harvested vessel, and modifying the risk factors of coronary artery disease with optimized therapy and lifestyle changes [15].

The CHA_2_DS_2_-VASc score was first described in the literature as a tool for predicting thromboembolic events in patients with AF [7]. Thereafter, since it is simple and feasible to use, the score was investigated for further use in cardiovascular outcomes. Rozenbaum et al. [16] investigated the association between CHA_2_DS_2_-VASc and acute coronary syndrome and found that a higher CHA_2_DS_2_-VASc score identified patients with a higher risk of the acute coronary syndrome. In another study, Kurtul et al. [17] found that increased CHA_2_DS_2_-VASc score was associated with increased mortality and increased coronary artery disease in patients with the acute coronary syndrome. Furthermore, besides coronary artery disease, the outcomes of prosthetic valvular diseases were also studied in the literature. Çınar et al. [18] investigated the association between CHA_2_DS_2_-VASc score and prosthetic mechanical valve thrombosis and found that the CHA_2_DS_2_-VASc score might be an independent predictor of prosthetic valve thrombosis. Because the CHA_2_DS_2_-VASc score includes risk factors for atherosclerotic cardiovascular disease among its parameters, the score can also represent the thrombotic and atherosclerotic burden of the individuals.

In our study, DM, hypertension, HFrEF, and an increase in creatinine levels were significantly associated with GF. Each parameter was associated with increased atherosclerosis and cardiovascular risk [19-21]. Kubiak et al. [22] investigated atherosclerosis of saphenous grafts with optical coherence tomography and found that decreased ejection fraction and renal insufficiency were associated with increased atherosclerosis in saphenous grafts. Their finding was similar to the findings of our study, where GF was significantly associated with HFrEF and increased creatinine level. However, the creatinine levels were in the normal range for both groups in our study. Additionally, the information in the literature regarding the role of hypertension and DM in graft patency is controversial. Muir et al. [13] concluded that either hypertension or DM contributes to graft patency after CABG surgery. On the contrary, Goldman et al. [2] found that neither hypertension nor DM contributes to graft patency. In our study, these parameters were strongly associated with GF; however, none of them proved to be an independent predictor of GF in multivariate analysis. Furthermore, only the CHA_2_DS_2_-VASc score was found to be an independent predictor of GF. This finding proves that each parameter might not predict GF individually, but with a simple and feasible calculation method, which includes these risk factors, the incidence of GF might be predicted.

A study similar to ours by Yarlioglues et al. [23] investigated the association between the CHA_2_DS_2_-VASc score and saphenous vein graft disease. They found that a CHA_2_DS_2_-VASc score ≥4 was an independent predictor of saphenous vein graft disease. However, there were some differences between their study and our analysis. In their study, they also found that time interval after CABG was an independent predictor of saphenous vein graft disease, and their data had significant time difference in the intervals after CABG between the studied groups: patients with saphenous vein graft disease had longer time interval after CABG surgery. Their findings among study groups might have been influenced by this significant difference. In our study, the time interval after CABG between the groups was similar and there was no statistically significant difference in terms of left heart catheterization (LHC) graft study between groups. Another difference was that they had excluded arterial grafts; however, our study included both saphenous vein grafts and internal mammary artery grafts. Additionally, their study defined saphenous vein graft disease as the presence of at least 50% stenosis in the grafts and they evaluated the stenosis with visual assessment. In our study, we did not include moderate degeneration in saphenous vein grafts and defined graft failure as stenosis ≥70%, and these segments with stenosis were evaluated not only by visual assessment but also with QCA analysis. Therefore, our study addressed most of the limitations in their study and had more precise findings compared to their study.

Study limitations

There were several limitations to this study. Firstly, because the parameters were identified retrospectively, this study is prone to limitations inherent to any retrospective analysis. Secondly, this was a single-center study with a relatively small sample size, which is a limitation in that the findings cannot be representative of the broader population. In addition, we could not separately include patients with acute angina pectoris because of the small number of patients. Finally, the follow-up period was 100 months on average and we did not have the data on longer-term outcomes after surgery in our study.

## Conclusions

Based on our findings, graft patency remains a significant challenge after CABG surgery. The CHA_2_DS_2_-VASc score might be used as a handy, feasible, and simple tool to predict the risk of GF following CABG surgery. Thus, patients with high CHA_2_DS_2_-VASc scores might be followed up closely. However, more comprehensive studies should be conducted on this subject to validate our findings.
